# Automated Detection of Driver Fatigue Based on AdaBoost Classifier with EEG Signals

**DOI:** 10.3389/fncom.2017.00072

**Published:** 2017-08-03

**Authors:** Jianfeng Hu

**Affiliations:** The Center of Collaboration and Innovation, Jiangxi University of Technology Nanchang, China

**Keywords:** driver fatigue, electroencephalogram (EEG), adaboost, fuzzy entropy, receiver operating characteristic (ROC)

## Abstract

**Purpose:** Driving fatigue has become one of the important causes of road accidents, there are many researches to analyze driver fatigue. EEG is becoming increasingly useful in the measuring fatigue state. Manual interpretation of EEG signals is impossible, so an effective method for automatic detection of EEG signals is crucial needed.

**Method:** In order to evaluate the complex, unstable, and non-linear characteristics of EEG signals, four feature sets were computed from EEG signals, in which fuzzy entropy (FE), sample entropy (SE), approximate Entropy (AE), spectral entropy (PE), and combined entropies (FE + SE + AE + PE) were included. All these feature sets were used as the input vectors of AdaBoost classifier, a boosting method which is fast and highly accurate. To assess our method, several experiments including parameter setting and classifier comparison were conducted on 28 subjects. For comparison, Decision Trees (DT), Support Vector Machine (SVM) and Naive Bayes (NB) classifiers are used.

**Results:** The proposed method (combination of FE and AdaBoost) yields superior performance than other schemes. Using FE feature extractor, AdaBoost achieves improved area (AUC) under the receiver operating curve of 0.994, error rate (ERR) of 0.024, Precision of 0.969, Recall of 0.984, F1 score of 0.976, and Matthews correlation coefficient (MCC) of 0.952, compared to SVM (ERR at 0.035, Precision of 0.957, Recall of 0.974, F1 score of 0.966, and MCC of 0.930 with AUC of 0.990), DT (ERR at 0.142, Precision of 0.857, Recall of 0.859, F1 score of 0.966, and MCC of 0.716 with AUC of 0.916) and NB (ERR at 0.405, Precision of 0.646, Recall of 0.434, F1 score of 0.519, and MCC of 0.203 with AUC of 0.606). It shows that the FE feature set and combined feature set outperform other feature sets. AdaBoost seems to have better robustness against changes of ratio of test samples for all samples and number of subjects, which might therefore aid in the real-time detection of driver fatigue through the classification of EEG signals.

**Conclusion:** By using combination of FE features and AdaBoost classifier to detect EEG-based driver fatigue, this paper ensured confidence in exploring the inherent physiological mechanisms and wearable application.

## Introduction

Electroencephalogram (EEG) is a very important monitoring technique to reflect the instantaneous state of the brain. Various computational ways based on EEG signals have been successfully used to assist the diagnosis of seizure (Amal Feltane et al., 2013), stroke, Alzheimer's, schizophrenia (Boostani et al., 2009), epilepsy (Guo et al., 2010), depression, Attention Deficit Hyperactivity Disorder, and even fatigue. Driver fatigue is very important factor to traffic safety and automated detection is necessary urgently (Lal and Craig, 2001). Many EEG-based studies have been performed to analyze and detect driving fatigue (Kar et al., 2010; Mu et al., 2017a; Yin et al., 2017).

Correa et al. got 83.6% accuracy using a Neural Network classifier (Correa et al., 2014). Mousa Kadhim et al. yielded the highest accuracy of 85% using Discrete Wavelet Transforms method (Mousa Kadhim et al., 2013). Li et al. achieved the highest accuracy of 91.5% based on 12 types of energy parameters (Li et al., 2012). Fu et al. reached a highest accuracy of 92.5% based on Hidden Markov Model (HMM; Fu et al., 2016). Zhao et al. hit a higher accuracy (98.7%) based on a KPCA-SVM classifier (Zhao et al., 2010). Recently, entropy has been broadly applied in the analysis of EEG signals, considering the fact that EEG is a complex, unstable, and non-linear signal (Acharya et al., 2012; Hu et al., 2015; Mu et al., 2016). A diverse varied collection of these methods has been proposed in the last few decades, including spectral entropy (PE), permutation entropy, distribution entropy, fuzzy entropy (FE), Renyi entropy, approximate entropy (AE), sample entropy (SE), and some others. Specially, in the field of EEG processing, four of the most widely used and successful entropy estimators are FE (Chen et al., 2009), SE (Richman and Moorman, 2000), AE (Pincus, 1991), and PE (Reyes-Sanchez et al., 2016). AE has demonstrated its capability to detect complexity changes. SE is a similar statistic, which has not yet been used as extensively as AE. AE and SE are very successful entropy features, but they also have their weaknesses. AE is biased because it includes self-matching in the count, while SE needs to avoid the log(0) problem. They are also very sensitive to input parameters. More recently, FE has been proposed to alleviate these problems. FE is based on a continuous function to compute the dissimilarity between two zero-mean subsequences, so it is more stable in noise and parameter initialization.

Liu et al. got 84% accuracy with the combination of kernel principal component analysis and HMM utilizing AE and Kolmogorov complexity to detect the fatigue state (Liu et al., 2010). Mu et al. yielded accuracy of 85% with FE and Support Vector Machine (SVM) classifier (Mu et al., 2017a). Xiong et al. proposed a feature combination of AE and SE with SVM classifier to test driving fatigue, and achieved the best accuracy of 91.3% (Xiong et al., 2016). Khushaba et al. exploited a feature extraction by using fuzzy mutual-information method and achieved 92.8% (Khushaba et al., 2011). Hu hit highest accuracy of 96.6% with FE and Random Forest classifier (Hu, 2017).

From the literature review, it has been observed that few studies have been conducted for using ensemble classifier based on EEG to study driver fatigue detection. Keeping this in mind, the prime motivation of this work is to develop an automated detection system for driver fatigue based on ensemble classifier. The scheme employs four types of entropy for feature extraction and AdaBoost (Freund and Schapire, 1997; Hastie et al., 2009) for classification of EEG signals into normal and fatigue. Several experiments on 28 subjects indicate that the proposed scheme earns better detection performance and robustness in comparison to other existing schemes.

The rest of this article is described as below. In Materials and Methods, data acquisition, feature extraction, and classification are illustrated. The results are discussed in Section Results presents the evaluation of the method with the obtained results, followed by a general discussion about classifier accuracy in Section Discussion.

## Materials and methods

### Subjects

Twenty-eight university students (14 male, 19–24 years) participated in this experiment, which all had a current driver's license. Before the experiment, they practiced driving for several minutes to familiarize themselves with the process and purpose of the experiment. The experiment was approved by the Academic Ethics Committee of the Jiangxi University of Technology according to the standards of the Declaration of Helsinki. Written informed consent was obtained from each subject.

### Experiment

**Figure 1:**
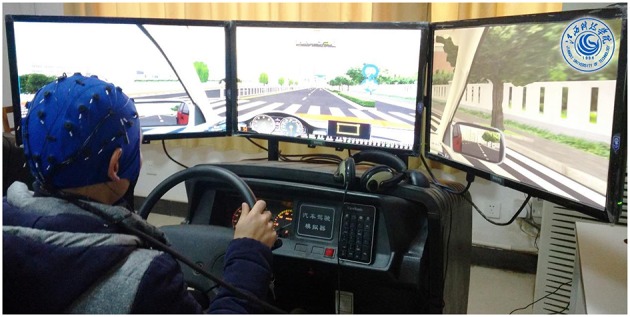
Snapshot of the experimental setup.

In the static driving simulator (ZY-31D, ZhongYu CO., LTD, China), the driver's fatigue simulation test was performed on each subject, as shown in Figure 1. The driving environment selected for this work was a highway with low traffic density so as to induce monotonous driving, which easily leads to driver fatigue state.

### Data recording

Similar to former experiments (Chai et al., 2017; Hu, 2017; Mu et al., 2017b), when the driving procedure started 20 min, the last 5-min EEG recordings were marked as normal state. When the continuous driving procedure lasted 60–120 min until the questionnaire results (Lee's subjective fatigue scale and Borg's CR-10 scale; Borg, 1990; Lee et al., 1991), participants' responses and electrooculogram (EOG) signals show the subject was in driving fatigue state, the last 5-min recorded EEG recordings were marked as fatigue state. EOG signals were used to determine fatigue state using the blink rate and eye closure such as, the small and slow blinks.

All channel data were referenced to two reference electrodes A1 and A2, and digitized at 1 kHz from a 32-channel electrode cap (including 2 reference electrodes) based on the international 10–20 system.

After the acquisition of EEG signals, the main procedures of data preprocessing was implemented by Scan 4.3 software of Neuroscan (Compumedics, Australia). The original signals were first filtered and a 0.15–45 Hz band-pass filter was used. Then 5-min EEG signals from 30 channels were sectioned into 1-s epochs, resulting in 300 epochs. With the 28 subjects and 30 channels, a total of 504,000 units were randomly formed for dataset (each state having 252,000 units).

### Feature extraction

The EEG is assumed to be a non-stationary time series and most feature extraction methods are only applicable to stationary signal. To deal with this problem, the EEG time series were divided into several short windows and its statistics is assumed to be approximately stationary within each window. The following feature extraction methods are applied to each 1-s windowed signal. EEG signals are segmented without overlap, finally feature sets are extracted from all channels in each 1 s window.

The ability to distinguish between normal state and fatigue state depends mainly on the quality of the input vectors of the classifier. In order to capture EEG features, four feature sets are computed, including FE, SE, AE, and PE. In this section, the computational methods of these feature sets in EEG recordings are described in detail.

#### Spectral entropy (PE)

PE is evaluated using the normalized Shannon entropy (Kannathal et al., 2005), which quantifies the spectral complexity of the time series. The power level of the frequency component is indicated by *Y*_*i*_ and *y*_*i*_ is normalized as:

(1) $$\begin{array}{r} y_{i}=\frac{Y_{i}}{\sum Y_{i}} \end{array}$$

The PE of the time series is calculated using the following equation:

(2) $$\begin{array}{r} PE=\sum iy_{i}\log\left(\frac{1}{y_{i}}\right) \end{array}$$

#### Approximate entropy (AE)

AE, as proposed by Pincus (1991), is a statistically quantified non-linear dynamic parameter that measures the complexity of a time series. The procedure for the AE-based algorithm is described as follows:

1. Considering a time series *t*(*i*), a set of m-dimensional vectors are obtained according to the sequence order of *t*(*i*):
  (3) $$\begin{array}{r} T_{i}^{m}=\left[t\left(i\right),t\left(i+1\right),\;\ldots ,\;t\left(i+m-1\right)\right];i\leq L-m+1 \end{array}$$
  $d\left[T_{i}^{m},T_{j}^{m}\right]$is the distance between two vectors $T_{i}^{m}$ and$T_{j}^{m}$, defined as the maximum difference values between the corresponding elements of two vectors:
  (4) $$\begin{array}{l} d[T_{i}^{m},T_{j}^{m}]=\underset{\;k\in (0,m-1)}{\text{max}\left\{\left|t(i+k)-t(j+k)\right|\right\},(i,j=1\sim L-m}\\ \;+1,i\neq j) \end{array}$$
2. Define *S*_*i*_ is the number of vectors *T*_*j*_ that are similar to*T*_*i*_, subject to the criterion of similarity $d\left[T_{i}^{m},T_{j}^{m}\right]\leq s$
  (5) $$\begin{array}{r} S_{i}^{m}\left(s\right)=\frac{1}{L-m+1}S_{i} \end{array}$$
3. Define the function γ^*m*^(*s*) as:
  (6) $$\begin{array}{r} \gamma^{m}\left(s\right)=\;\frac{1}{L-m+1}\overset{L-m+1}{\underset{i=1}{\sum }}\ln\;S_{i}^{m}\left(s\right) \end{array}$$
4. Set *m* = *m* + 1, and repeat steps (3) to (6) to obtain $S_{i}^{m+1}\left(s\right)$ and γ^*m*+1^(*s*), then:
  (7) $$\begin{array}{r} \gamma^{m+1}\left(s\right)=\;\frac{1}{L-m}\overset{L-m}{\underset{i=1}{\sum }}\ln\;S_{i}^{m+1}\left(s\right) \end{array}$$
5. The AE can be expressed as:
  (8) $$\begin{array}{r} AE=\;\gamma^{m}\left(s\right)-\;\gamma^{m+1}\left(s\right) \end{array}$$

#### Sample entropy (SE)

SE's algorithm is similar to that of AE (Yentes et al., 2013), which is a new measure of time series complexity proposed by Richman and Moorman (2000). The step (1) can be defined in the same way as the AE-based algorithm; other steps in the SE-based algorithm are described as follows:

1. Define *A*_*i*_ is the number of vectors *T*_*j*_ that are similar to *T*_*i*_ subject to the criterion of similarity $d\left[T_{i}^{m},T_{j}^{m}\right]\leq s$
  (9) $$\begin{array}{r} A_{i}^{m}\left(s\right)=\frac{1}{L-m-1}A_{i} \end{array}$$
2. Define the function γ^*m*^(*s*) as:
  (10) $$\begin{array}{r} \gamma^{m}\left(s\right)=\frac{1}{L-m}\overset{L-m}{\underset{i=1}{\sum }}A_{i}^{m}\left(s\right) \end{array}$$
3. Set *m* = *m* + 1, and repeat above steps to obtain $A_{i}^{m+1}\left(s\right)$ and γ^*m*+1^(*s*), then
  (11) $$\begin{array}{r} \gamma^{m+1}\left(s\right)=\frac{1}{L-m-1}\overset{L-m-1}{\underset{i=1}{\sum }}A_{i}^{m+1}\left(s\right) \end{array}$$
4. The SE can be expressed as:
  (12) $$\begin{array}{r} SE=log\left(\gamma^{m}\left(s\right)/\gamma^{m+1}\left(s\right)\right) \end{array}$$

#### Fuzzy entropy (FE)

To deal with some of the issues with SE, Xiang et al. proposed the use of fuzzy membership function in computing the vector similarity to replace the binary function in SE algorithm (Xiang et al., 2015), so that the entropy value is continuous and smooth. The procedure for the FE-based algorithm is described in detail as follows:

1. Set a *L*-point sample sequence: {*v*(*i*):1 ≤ *i* ≤ *L*};
2. The phase-space reconstruction is performed on *v(i)* according to the sequence order. The reconstructed vector can be written as:
  (13) $$\begin{array}{r} T_{i}^{m}=\left\{v\left(i\right),v\left(i+1\right),\ldots ,v\left(i+m-1\right)\right\}-v_{0}\left(i\right) \end{array}$$
  where *i* = 1, 2, …, *L* − *m* + 1, and *v*_0_(*i*) is the average value described as the following equation:
  (14) $$\begin{array}{r} \upsilon_{0}\left(i\right)=\;\frac{1}{m}\overset{m-1}{\underset{j=0}{\sum }}\upsilon \left(i+j\right) \end{array}$$
3. $d_{ij}^{m}$, the distance between two vectors $T_{i}^{m}$ and$T_{j}^{m}$, is defined as the maximum difference values between the corresponding elements of two vectors:
  (15) $$\begin{array}{l} d_{ij}^{m}=\text{d}\left[T_{i}^{m},T_{j}^{m}\right]=\max_{k\in (0,m-1)}\{|\upsilon \left(i+k\right)-\upsilon_{0}\left(i\right)\\ \;-(\upsilon \left(j+k\right)-\upsilon_{0}\left(j\right))|\}\\ \left(i,j=1\sim L-m,i\neq j\right) \end{array}$$
4. According to the fuzzy membership function $\sigma \left(d_{ij}^{m},n,s\right)$, the similarity degree $D_{ij}^{m}$ between two vectors $T_{i}^{m}$ and $T_{j}^{m}$ is defined as:
  (16) $$\begin{array}{r} D_{ij}^{m}=\sigma \left(d_{ij}^{m},n,s\right)=\exp\left(-{\left(d_{ij}^{m}\right)}^{n}/s\right) \end{array}$$
  where the fuzzy membership function $\sigma \left(d_{ij}^{m},n,s\right)$ is an exponential function, while *n* and *s* are the gradient and width of the exponential function, respectively.
5. Define the functionγ^*m*^(*n, s*):
  (17) $$\begin{array}{r} \gamma^{m}(n,s)=\frac{1}{L-m}\sum_{i=1}^{L-m}\frac{1}{L-m-1}\sum_{j=1,j\neq 1}^{L-m}D_{ij}^{m}] \end{array}$$
6. Repeat the steps from (1) to (4) in the same manner. Define the function:
  (18) $$\begin{array}{l} \gamma^{m+1}(n,s)=\frac{1}{L-m}\sum_{i=1}^{L-m}\frac{1}{L-m-1}\sum_{j=1,j\neq 1}^{L-m}D_{ij}^{m+1}] \end{array}$$
7. The FE can be expressed as:
  (19) $$\begin{array}{r} \text{FE}\left(\text{m},\text{ s},\text{n}\right)=ln\gamma^{m}\left(n,s\right)-ln\gamma^{m+1}\left(n,s\right) \end{array}$$

In these four entropies, *m* and *s* are the dimensions of phase space and similarity tolerance, respectively. In the present study, *m* = 2, *n* = 4 while *s* = 0.2 ^*^ *SD*, where *SD* denotes the standard deviation of the time series.

For optimizing the detection quality, the feature sets were normalized for each subject and each channel by scaling between −1 and 1.

### Classification

To avoid over-fitting problem, the datasets were separated into train sets and test sets in the following pattern. In the train phase, 10-fold cross validation applied on the features such that 10% feature vectors are dedicated as test set and other 90% feature vectors are considered as the train set. In the next iteration, another 10% feature vectors consider as test set and the rest for the train set, till all of feature vectors involved one time in the test process. The final result was obtained by averaging the results of corresponding turns. By this evaluation scheme, the dependencies of the train and test sets were eliminated.

Since there is no uniform classification method suitable for all subjects and all applications, usually it may be useful to test multiple methods (Zhang et al., 2017). In this work, three types of base classifiers namely Decision Trees (DT), Support Vector Machine (SVM), and Naive Bayes (NB) were used. DT is a non-parametric supervised learning method used for classification. DT establishes several binary decision functions on the features. DT1 and DT9 represent DT with the maximum depth of the tree being 1 and 9 in this work, respectively. In the case of non-linear classification, kernels, such as, radial basis functions (RBF), are used to map the data into a higher dimensional feature space in which a linear separating hyper-plane could be found. Naive Bayes method is based on applying Bayes' theorem with the “naive” assumption. The likelihood in NB of the features is assumed to be Gaussian. In this study, grid parameter search was used to achieve better results.

AdaBoost is an eminent ensemble learning based classification model (Amal Feltane et al., 2013; Yang et al., 2016), which was first proposed by Freund and Schapire (1997). AdaBoost produces the final output by weighting the decisions of all these weak classifiers using majority vote method. The AdaBoost algorithm is described as follow:

**Table:** 

**Algorithm** *AdaBoost*
*Definition train dataset (X, Y) = {(x_1_,y_1_), (x_2_,y_2_), (x_3_,y_3_), ……*, *(x_*N*_,y_*N*_)}, y_*i*_ ε {-1, 1}*
*iterator: M;*
*Initialize each weight* $W_{1,\;i}=\;\frac{1}{N}$*, i = 1, 2, ……, N*,
*Linear combination function of basic classifiers f*_0_(*x*) = 0
*for m = 1 to M do*
*train a base learner: D*_*m*_(*x*)
*calculate error rate:* $e_{m}=\;\overset{N}{\underset{i=1}{\sum }}W_{m,i}I\left(D_{m}\left(x_{i}\right)-\;y_{i}\right)$
$\alpha_{m}=\;\frac{1}{2}ln\left(\frac{1-e_{m}}{e_{m}}\right)$
*update weight:* $W_{m+1,\;i}=\;\frac{W_{m,\;i}}{Z_{m}}\;exp\;\left(-\alpha_{m}y_{i}D_{m}\left(x_{i}\right)\right),$
$normalization\;factor\;Z_{m}=\;\overset{N}{\underset{i=1}{\sum }}W_{m,i}\exp\left(-\alpha_{m}y_{i}D_{m}\left(x_{i}\right)\right)$
*f*_*m*_(*x*) = *f*_*m*−1_ (*x*) + *lr* * α_*m*_*D*_*m*_(*x*)
*end for*
*Output* $sign\left(f_{M}\left(x\right)\right)=sign\left(\overset{M}{\underset{m=1}{\sum }}\alpha_{m}D_{m}\left(x\right)\right)$

In this work, the DT9 was used as base classifiers.

### Performance evaluation

To provide an easier-to-understand method to assess the classification quality, the results of classification and the performance of classifiers are expressed in terms of Error rate, Precision, Recall, F1 score, MCC, and AUC which are defined as follows:

Error rate (ERR) calculates the total number of EEG segments which are incorrectly classified

$$\begin{array}{l} \text{ERR}=\frac{\left(\text{FP }+\text{ FN}\right)}{\left(\text{FP }+\text{ TN }+\text{ FP }+\text{ FN}\right)} \end{array}$$

The precision intuitively reflects the ability of the classifier to determine the whole sample—which the positive is identified as positive and the negative is identified as negative.

$$\begin{array}{l} \text{Precision}=\frac{TP}{TP+FP} \end{array}$$

The recall intuitively reflects the proportion of positive samples that are correctly identified.

$$\begin{array}{l} \text{Recall}=\frac{TP}{TP+FN} \end{array}$$

The F1 score can be interpreted as a weighted average of precision and recall, where an F1 score reaches the optimum value at 1 and the worst score at 0.

$$\begin{array}{l} \text{F}1=\frac{2\;*\;\left(\text{Precision }*\text{ Recall}\right)}{\left(\text{Precision }+\text{ Recall}\right)} \end{array}$$

The Matthews correlation coefficient (MCC) is used in machine learning as a measure of the quality of two-class classifications. The MCC is in essence a correlation coefficient value between -1 and +1.

$$\begin{array}{l} \text{MCC}=\frac{TP*TN-FP*FN}{\sqrt{\left(TP+FP\right)\left(TP+FN\right)\left(TN+FP\right)\left(TN+FN\right)}} \end{array}$$

Where

TP (True Positive) = correctly identified normal segments

TN (True Negative) = correctly identified fatigue segments

FN (False Negative) = incorrectly identified normal segments

FP (False Positive) = incorrectly identified fatigue segments

AUC illustrates the performance of a binary classifier system as its discrimination threshold is varied. It is created by drawing true positive rates from positive (true positive rate) and false positive rates (false positive rates) in a variety of threshold settings.

### Statistical analysis

In order to investigate differences of average accuracy among various classifiers and feature sets, the paired sample *t*-test was used to evaluate effectiveness on each comparison. The results are averages over 10 independently turns of combination of train set and test set in each experiment.

## Results

In order to verify the validity, effectiveness, and robust of proposed method, some experiments were performed on 28 subjects.

### Comparison with different feature sets and different classifiers

**Figure 2:**
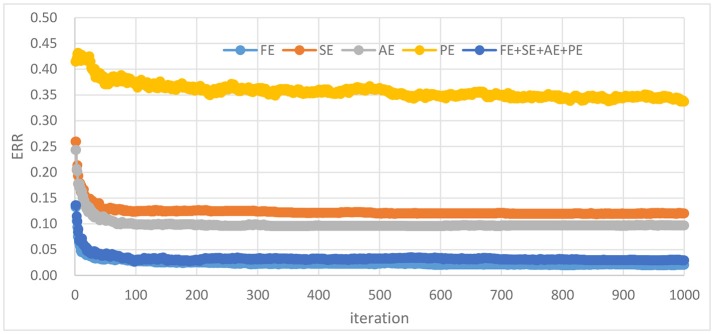
ERR for AdaBoost based on different feature sets.

As shown in Figure 2. FE feature set performs slightly better than the combined entropy (FE + SE + AE + PE) feature set (0.020 against 0.029). A paired *t*-test across the 10 independent comparisons indicates a significant difference with *p*-value around 0.003. It can be seen that the FE feature set performs about 0.098 and 0.075 better than the SE and AE feature set at ERR index, respectively. A paired *t*-test over 10 independent comparisons shows a significant difference with *p*-value lower than 0.001. AE feature set performs slightly better than SE feature set. It can also show that the PE feature set performs worst with the lowest ERR being about 0.337.

**Figure 3:**
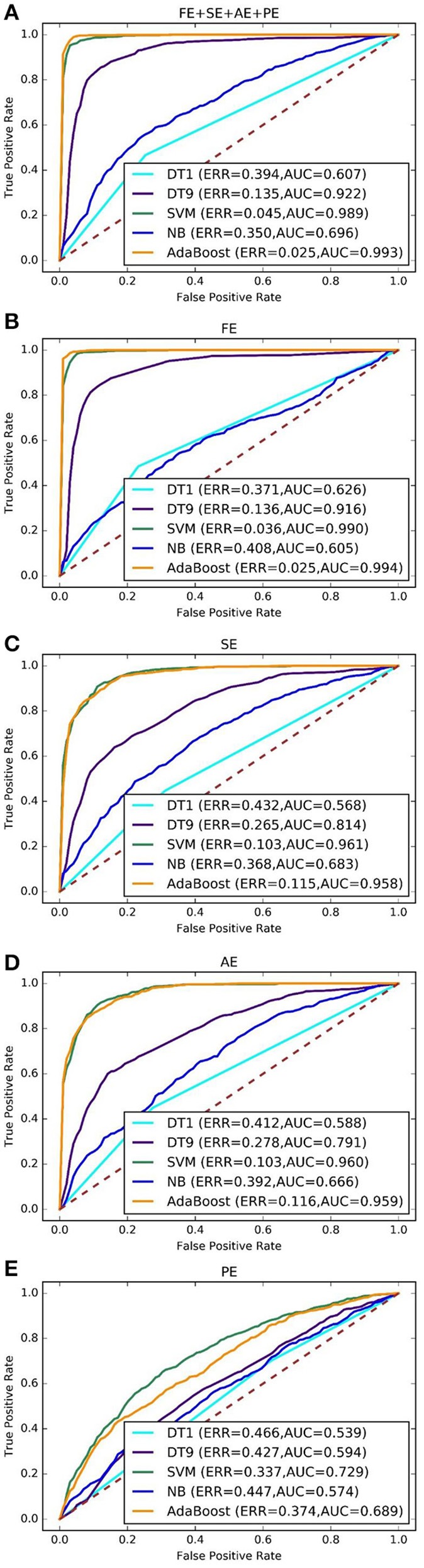
ROC curves for different feature sets and different classifiers. **(A–E)** Represents combined feature set, FE feature set, SE feature set, AE feature set and PE feature set, respectively.

The results of 10 independently rounds are used to draw mean ROC curves. Different feature sets or classifiers were compared by analyzing their ROC curves and areas under ROC curves (AUC). In Figures 3A–E, their performance in ROC curves produced was compared by different classifiers on combined entropy feature set, FE feature set, SE feature set, AE feature set and PE feature set, respectively. It shows that the FE feature set and combined feature set outperform other feature sets, which similar to Figure 2. For example, the best ERR of FE feature set and combined feature set are both 0.025, while the best ERR of SE, AE, and PE are 0.115, 0.116, and 0.374, respectively. The best AUC of FE feature set and combined feature set is are 0.993 and 0.994, respectively while the best ERR of SE, AE, and PE are 0.961, 0.960, and 0.729, respectively. Consequently, adding more features makes nothing changes for driver fatigue detection. Therefore, the FE feature set is selected for the next experiments.

**Figure 4:**
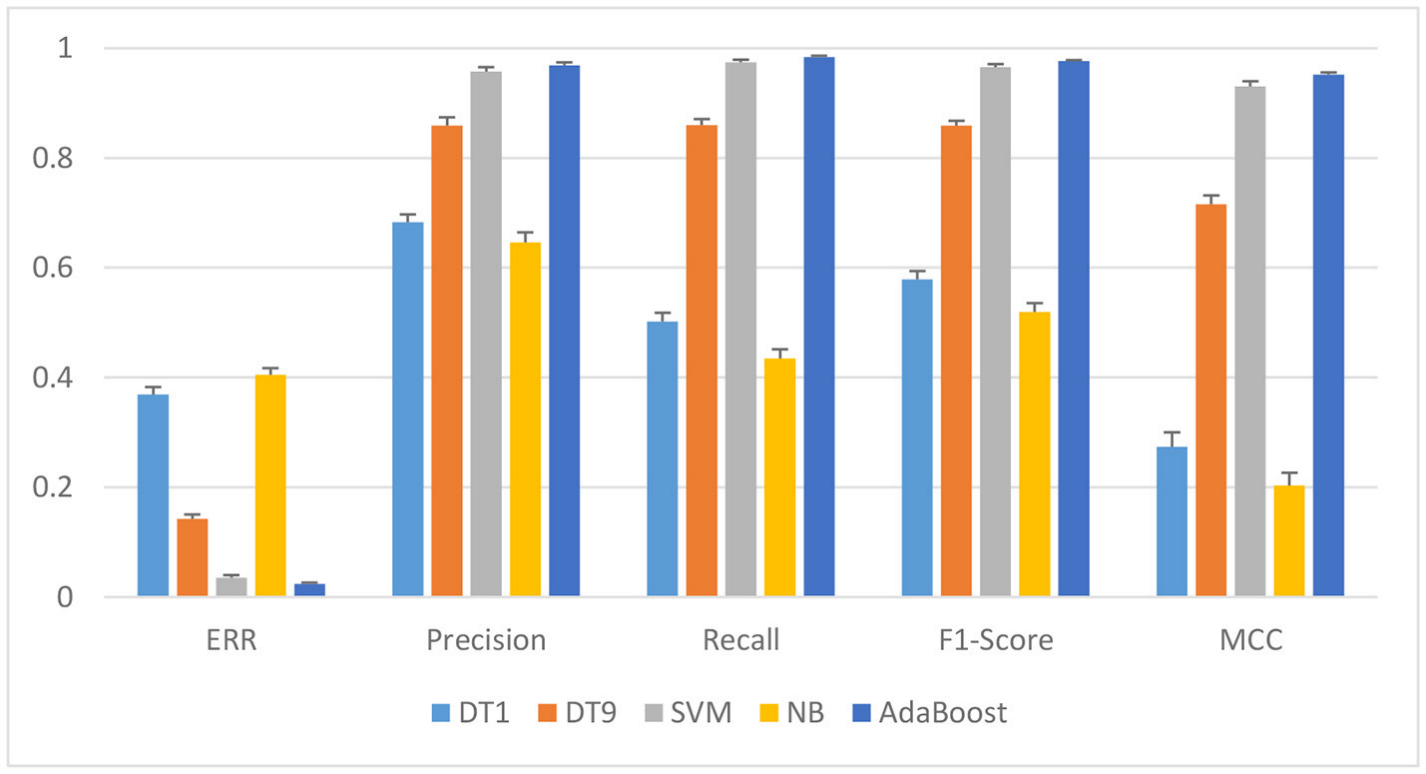
Performance of different classifiers based on FE feature set.

As shown in Figure 4, it illustrate that AdaBoost outperform other classifiers. For instance, the best ERR and AUC is 0.025 and 0.994 for AdaBoost based on FE feature set, while the best ERR and AUC is 0.036 and 0.990 for SVM based on FE feature set. The *p*-value is 0.0062 between AdaBoost and SVM. AdaBoost classifier is significantly better than other classifiers. The *p*-values are 0.0032 and 0.0001, by paired *t*-test between DT9 and AdaBoost, and between NB and AdaBoost, respectively. It's conjectured that AdaBoost models work best because they may be more robust than other models such as, DT and NB when dealing with scalar data sets that are not too larger.

To evaluate the effectiveness of AdaBoost in the classification of EEG signals, the classification indexes including ERR, Precision, Recall, F1, score and MCC of the four classifiers were compared based on FE feature set. As shown in Figure 4 and Table 1, the overall performance of AdaBoost is the best of the four classifiers in terms of ERR, Precision, Recall, MCC, and F1 score. The ERR of AdaBoost can reach 0.024 ± 0.002, which is almost 0.011 lower than SVM (0.035 ± 0.005). The ERR of DT1, DT9, and NB is 0.0369 ± 0.014, 0.0142 ± 0.008, and 0.405 ± 0.012, respectively.

**Table 1:** *p*-value between AdaBoost and other classifiers with paired *t*-test.

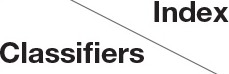	**ERR**	**Precision**	**Recall**	**F1 score**	**MCC**
SVM	1.05e-5	4.81e-4	5.60e-4	1.31e-4	8.92e-5
DT1	2.12e-7	4.82e-7	4.10e-7	4.46e-7	4.41e-7
DT9	2.07e-7	1.32e-6	9.46e-7	6.61e-7	6.10e-7
NB	2.06e-7	5.33e-7	3.98e-7	4.27e-7	3.97e-7

### Parameter setting

**Figure 5:**
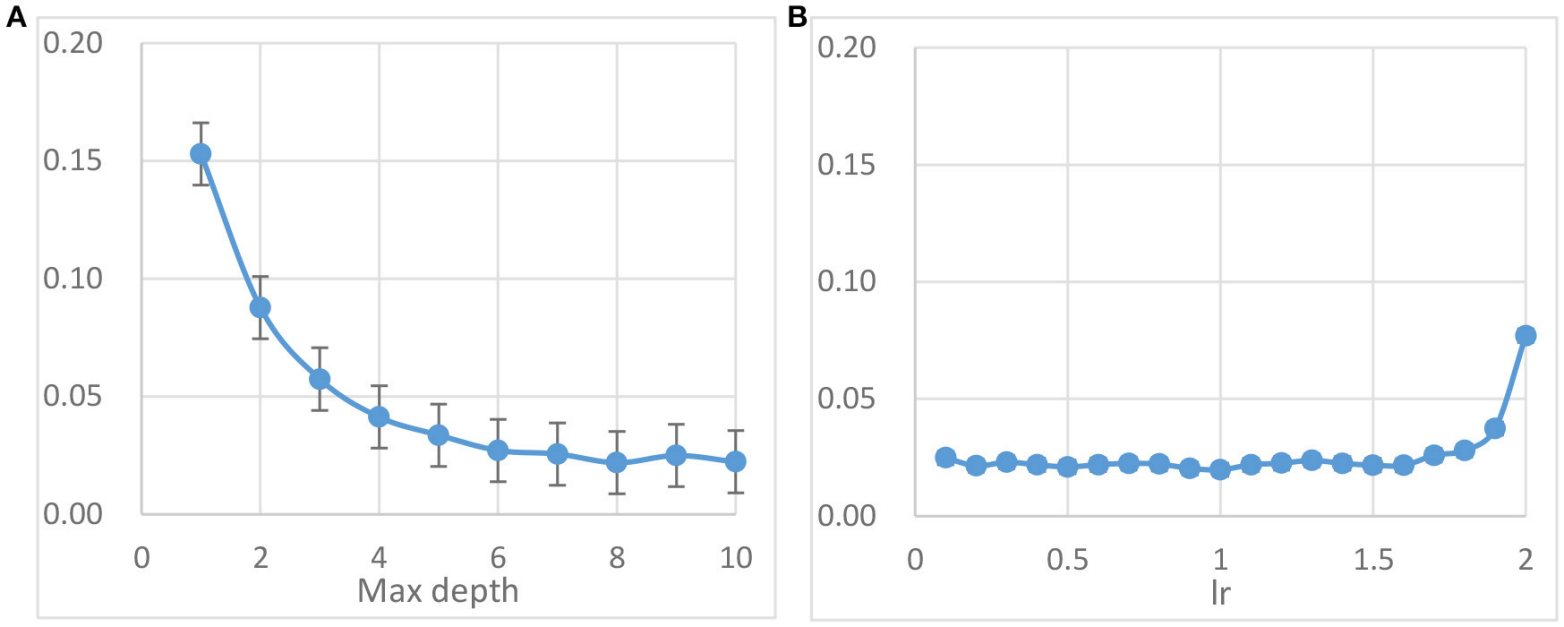
AdaBoost method parameter tuning results based on FE feature set and DT base classifier. **(A)** The error rates for different *max_depth* with *lr* = 1.0. **(B)** The error rates with default *max_depth* (value = 9) for different *lr*.

The main parameters to be adjusted in AdaBoost method are parameter *max_depth* of base classifier DT and *lr*. Best performance of AdaBoost model can be yielded through carefully choosing the optimal combination of these parameters. The parameter *max_depth* is the most important one in the DT, which controls the maximum depth of the tree. Figure 5A shows the error rates under different *max_depth* and fixed iteration (= 500) based on FE feature set. It is showed that the average error rate attains the minimal point 0.022 ± 0.004 when *max_depth* equals to about 8. From Figure 5B, it can be seen that the average error rate starts to even out at 0.022 ± 0.003 when the value of *lr* smaller than about 1.5. According to these results, the final AdaBoost classifier in next experiments is set with the parameters *max_depth* = 9 (DT9) and *lr* = 1.0.

### Comparison with different size of test samples

**Figure 6:**
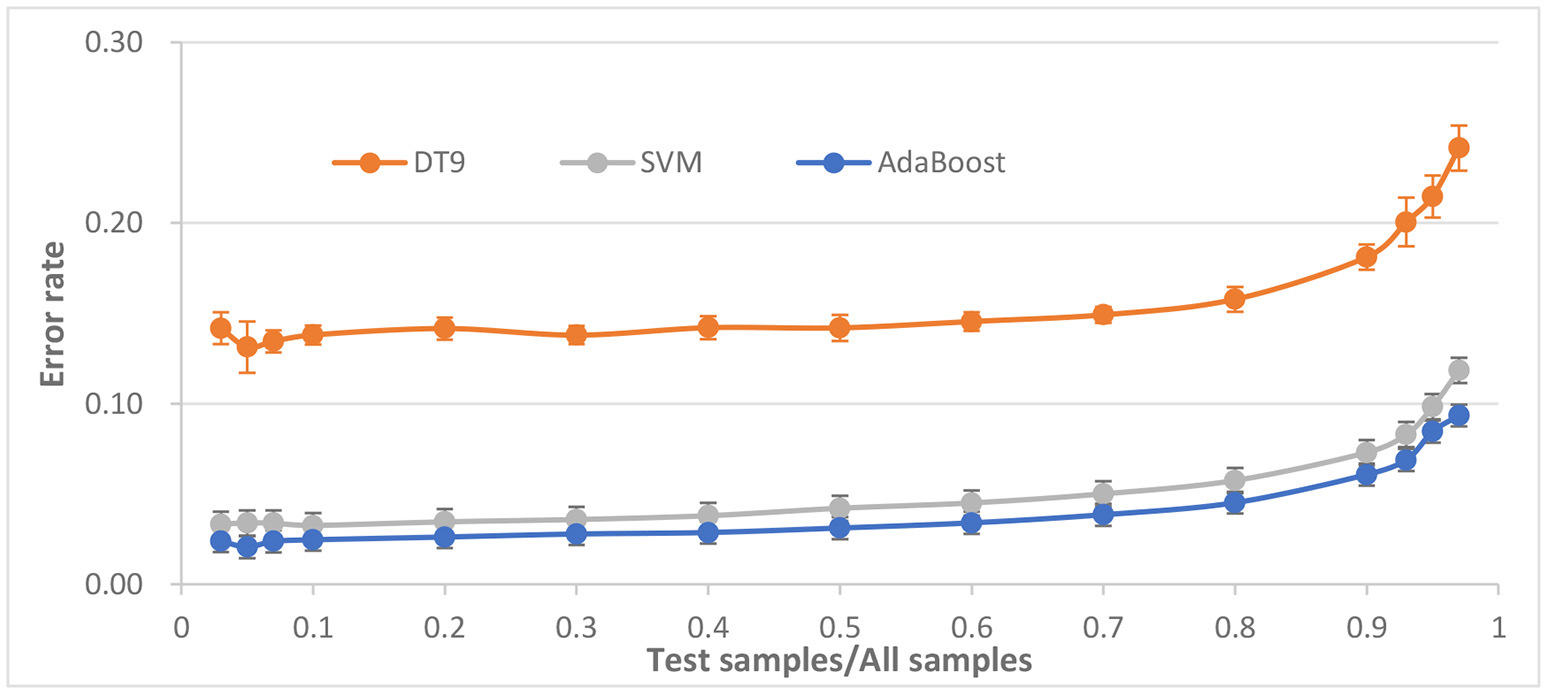
Performance evaluation with respect to the ratio of test samples for all samples.

The ratio of train samples for test samples is important for the performance of classifier. To determine the robustness of the classifier against size of test sample or train size, the ratio of test samples for all samples is set varying from 0.03 to 0.97. The ERR of AdaBoost against different ratio is shown in Figure 6.

It is observed that the average error rate begins to stabilize at about 0.03 when the ratio being about 0.5. When the ratio becomes larger, the ERR also becomes larger, but when ratio reaches close to 1.0, the ERR is close to 10% and becomes worse, possibly because of the lack of training samples. On the contrary, when the ratio becomes smaller, ERR is stable at around 0.02, which indicating that ratio is more appropriate in the 0.1.

*P*-value between AdaBoost and SVM, between AdaBoost and DT9, are 3e-8 and 4e-16, respectively. Compared to SVM and DT, AdaBoost seems to have better robustness against changes of ratio of test samples for all samples.

### Comparison with different number of subjects

The number of subjects is also an important parameter in the driving fatigue detection system. More subjects can provide more information that may improve or reduce detection performance. Generally speaking, when average performance is poor, any subject with higher accuracy can improve the overall performance, and vice versa. Sometimes, the classifier model that is suitable for small samples may lose performance when large samples are used. However, when more subjects are involved, the system costs, including hardware and computation time, will also increase. Therefore, a tradeoff between the system performance and system cost should be based on the specificity of the application.

To answer the question of how many subjects are needed to train for a satisfactory detection system, system performance was evaluated with respect to the number of subjects. For each number *n* (from 2 to 28), a random combination (*n* out of 28 subjects) was repeated 20 times for calculating classification accuracy using 10-fold cross validation. Three classifiers approaches were calculated for comparison. Furthermore, for each condition (*n* from 2 to 28), the paired *T*-test was used as a post-hoc test to evaluate if the performance of AdaBoost was significantly better than that of other two classifiers.

**Figure 7:**
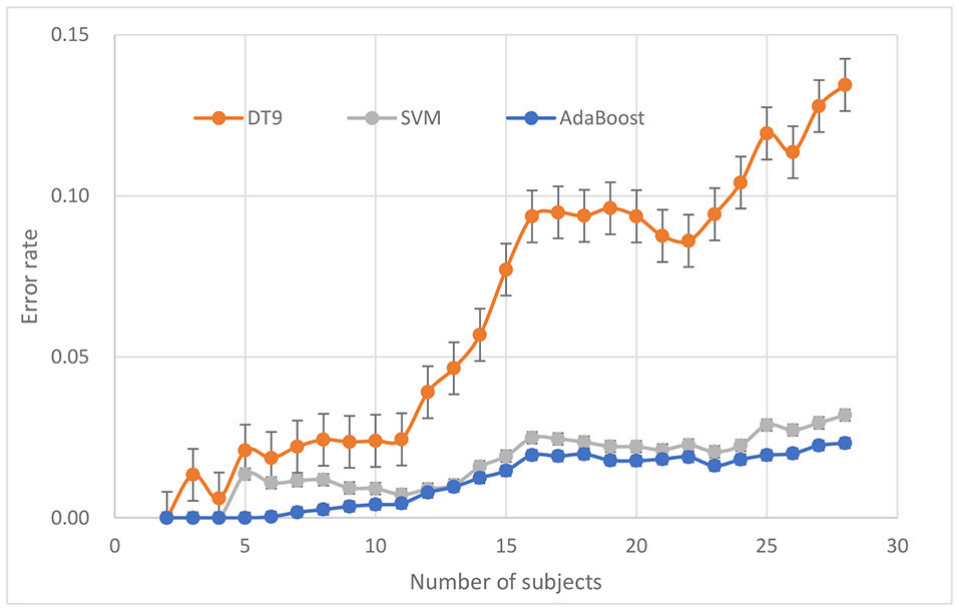
Performance evaluation in terms of number of subjects.

The ERR of AdaBoost against different number of subjects is shown in Figure 7. It can be seen that, for AdaBoost classifier, when the number of subjects is <13, ERR is <0.01, when the number of subjects continue to increase, ERR also increases, and is stable at about 0.02. ERR is not increasing monotonically with the number of subjects but tending to reach equilibrium.

*P*-values between AdaBoost and SVM, between AdaBoost and DT9, are 4.665e-8 and 1.409e-8, respectively. Compared to SVM and DT, AdaBoost seems to have better robustness against changes of number of subjects.

## Discussion

As see in Table 2, it is found that the classification performance of proposed method was better than that in the others research using entropy feature sets. Although, based on the existing EEG data, the optimal performance of detection of driving fatigue by using AdaBoost-based method showed well application on the real-time detection of driving fatigue.

**Table 2:** Studies regarding driver fatigue detection using entropy feature sets.

**Research group**	**Feature method**	**Highest accuracy (%)**
Liu et al., 2010	AE and others	84
Mu et al., 2017a	FE	85
Xiong et al., 2016	AE and SE	91.3
Khushaba et al., 2011	FE	92.8
Hu, 2017	FE	96.6
This paper	FE	97.5

Among the state-of-art classifier schemes, four representive algorithms, DT, NB, SVM, and AdaBoost were experimented for classification tasks of some data sets. These classifiers have been shown very effective in many pattern recognition applications. These classifiers are applied on the extracted features and their results are shown in Figures 3, 4 in which AdaBoost showed a better results in comparison with the three other classifiers.

Also to evaluate robustness of the classifiers, different combinations of train set and test sets were employed and the classification results were brought in Figures 6, 7. A repeated progressive method with various sample sizes was applied to find out if there is any relationship between data set size and the performance. It can be seen that AdaBoost is also more robust than the three other classifiers.

The experiment confirmed that, in comparison with the AE, PE, and SE, the FE had a better consistency and better discrimination ability. The results also showed that the differences between the normal state and the fatigue state were relative larger from the FE from the AE, SE, or the PE, confirming that the FE had a better performance in distinguishing fatigue state. The result achieved in this study ensured confidence in probing the theoretical reason for the different discrimination ability and, hence, leads to new ideas for exploring the inherent physiological mechanisms when using the entropy methods. This indicated that the FE could be an effective method for the driver fatigue detection.

However, there are several limitations in this study. First, it is worth noting that the parameter settings for the SE, AE, and PE method are the local similarity and parameters may not be the optimal solution. Second, the number of subject is relatively small. Although according to the existing literature in the Introduction section, the 28 subjects are not too small, but the number still needs to be increased. Third, only three commonly used classifiers and the four feature sets were compared in this study. Last, the different impacts of different channels haven't been took into account.

## Conclusion

In this paper, a method to develop an ensemble classifier for recognizing fatigue was proposed. A new EEG feature vector based on FE, SE, AE, and PE was used as input into four different classifiers: DT, NB, SVM, and AdaBoost. It was concluded that the combination of these feature sets or FE feature set with the AdaBoost provided the best performance on EEG dataset. The proposed method had very high accuracy classifying driver fatigue events. Further, it was showed how the method for detecting fatigue segments was robust.

## Author contributions

JH conceived and designed the experiments; JH analyzed the data and wrote the paper.

### Conflict of interest statement

The author declares that the research was conducted in the absence of any commercial or financial relationships that could be construed as a potential conflict of interest.

## Abbreviations

FE: Fuzzy entropy
SE: Sample entropy
AE: Approximate entropy
PE: Spectral entropy
EEG: Electroencephalogram
AUC: Areas under ROC curves
SVM: Support Vector Machine
DT: Decision Tree
NB: Naive Bayes
AdaBoost: Adaptive Boosting
ERR: Error rate.
